# Comprehensive analysis of grazing intensity impacts alpine grasslands across the Qinghai-Tibetan Plateau: A meta-analysis

**DOI:** 10.3389/fpls.2022.1083709

**Published:** 2023-01-17

**Authors:** Zhenchao Zhang, Yiran Zhao, Hao Lin, Yanpeng Li, Jinmin Fu, Yingxin Wang, Juan Sun, Yanhua Zhao

**Affiliations:** ^1^ Key Laboratory of National Forestry and Grassland Administration on Grassland Resources and Ecology in the Yellow River Delta, College of Grassland Science, Qingdao Agricultural University, Qingdao, Shandong, China; ^2^ School of Mapping and Geographic Information, Jiangxi College of Applied Technology, Ganzhou, China; ^3^ Grassland Research Center of National Forestry and Grassland Administration, Research Institute of Ecological Conservation and Restoration, Chinese Academy of Forestry, Beijing, China

**Keywords:** grazing intensity, ecosystem function, alpine grassland, Qinghai-Tibetan Plateau, meta-analysis

## Abstract

Livestock grazing is a dominant practice in alpine grasslands and plays a crucial role in the ecosystem service of the Qinghai-Tibetan Plateau. The effects of grazing on alpine grasslands highly depends on grazing intensity. Up to now, we still lack comprehensive understanding of the general responses of alpine grasslands to different grazing intensities over broad geographic scales across the Qinghai-Tibetan Plateau. Here, we conducted a meta-analysis to explore the responses of plant characteristics and soil properties to grazing intensity in alpine grasslands of the Qinghai-Tibetan Plateau based on 52 peer-reviewed literatures. The results showed that grazing did not change the belowground biomass, while significantly increased the ratio of root to shoot (*P<* 0.05). Light grazing exhibited no significant effects on the plant richness, Shannon-Wiener diversity, soil water content, soil bulk density, nutrients, microbial biomass carbon, and microbial biomass nitrogen (*P* > 0.05). Moderate grazing significantly increased the plant richness and Shannon-Wiener diversity, while significantly decreased the soil organic carbon and total nitrogen (*P<* 0.05). Heavy grazing significantly decreased the plant richness, Shannon-Wiener diversity, water content, soil organic carbon, total nitrogen, microbial biomass carbon, and microbial biomass nitrogen, and significantly increased the soil bulk density (*P<* 0.05). These findings suggest that overgrazing is closely associated with grassland degradation, and moderate grazing is a sustainable practice to provide animal production and simultaneously maintain ecological functions for alpine grasslands on the Qinghai-Tibetan Plateau.

## 1 Introduction

Grassland ecosystems are of multi-functionality which plays critical roles in supporting and regulating ecological processes including carbon sequestration, hydrological functions, and providing habitat for plants and animals (Eldridge and Delgado-Baquerizo, 2017; Yan et al., 2020). Herbivore grazing is a primary practice of the grasslands affecting multiple plant characteristics and soil properties of grasslands (Zhou et al., 2017; Sun et al., 2020; Liu et al., 2021). Due to the rapid economic development, there have been increasing demands on grasslands during recent decades (Kemp et al., 2013; Fetzel et al., 2017). The Qinghai-Tibetan Plateau whose main ecosystem is alpine grassland (Piao et al., 2012) occupies 2.5 × 10^8^ km^2^ and has a mean altitude of over 4000 m (Sun and Qin, 2016). The alpine ecosystem is fragile and extremely sensitive to grazing disturbance (Sun and Wang, 2016; Zhang Z. C. et al., 2021). Recently, alpine grasslands across the Qinghai-Tibetan Plateau have been suffering from grievous degradation as a result of the escalating impact from overgrazing (Cui and Graf, 2009; Zhao et al., 2016), seriously threatening the local ecological security and sustainable development. Therefore, there has been increasing attention paid to sustainable management of alpine grassland (Zhan et al., 2020; Sun et al., 2021a).

Large herbivores can exert profound effects on alpine grasslands by directly selective consumption and trampling (Sun et al., 2018; Zhong et al., 2022) as well as indirectly changing resource availability (Farji-Brener and Werenkraut, 2017; Zhong et al., 2021). On one hand, grazing reduces aboveground biomass by direct removal of phytomass which simultaneously increases ground-level light availability for shorter species (Borer et al., 2014; Ameztegui and Coll, 2015). Thus, grazing has been proven to promote plant diversity (Bai et al., 2004; Zhan et al., 2020). On the other hand, large herbivores generally reduced the organic matter input by directly removing of plant biomass (Lin et al., 2011; Deng et al., 2014). Due to less aboveground photosynthate allocation to root, grazing may possibly reduce belowground biomass (Bai et al., 2015). Moreover, the grazing-induced soil compaction by trampling limits root penetration and development (Sanjari et al., 2008). Meanwhile, the selective consumption of livestock increases the proportion of forbs with lower decomposability and thereby constrains the soil nutrient accumulation (Semmartin et al., 2010; Li et al., 2014; Zhang R. Y. et al., 2021). These likely reduce soil organic matter input and further decrease soil nutrient availability. However, previous studies found that grazing might actually increase plant biomass resulted from the grazing-induced compensatory effects (Bai et al., 2004; Niu et al., 2009). Moreover, grazing can lead to a biomass transfer from aboveground to belowground and thus benefit organic matter returned to the soil (De Deyn et al., 2008; Sun et al., 2021b). Additionally, the excretion input of large herbivores not only improves soil nutrient availability (Deng et al., 2014), but also promote soil microbial activities (McNaughton et al., 1997). Yet, the trampling of large herbivores enhances topsoil compaction, decreases porosity, and thus worsens water and aeration status of the soil, which inhibits soil microbial activities and causes decreases in soil quality and fertility (Holst et al., 2008; Chen et al., 2011).

It is well known that the various effects of grazing on alpine grasslands greatly depend on the grazing intensity (Steffens et al., 2010; Yang et al., 2016), whose roles may be varied considerably in alpine grasslands of the Qinghai-Tibetan Plateau compared with other rangeland ecosystems (Yang et al., 2021; Zhang et al., 2022). Comprehensive understanding of effects of grazing intensity on alpine grasslands is valuable to determine the optimal intensity for sustainable grazing in order to support alpine grassland management. Although the effects on grazing intensity on alpine grasslands have been widely reported in multiple studies, most of them are small-scale field studies (Yang et al., 2016; Deng et al., 2017; Zhan et al., 2020; Liu et al., 2021; Sun et al., 2021b). We still lack knowledge about the responses of alpine grasslands to different grazing intensities at a large scale across the Qinghai-Tibetan Plateau. This limits our ability to gain a better understanding of maintaining services of grazing ecosystems much less guiding sustainable management for the alpine grasslands. Here, we selected 52 peer-reviewed literatures to conduct a synthesis on responses of plant characteristics and soil properties to grazing intensity in alpine grasslands of the Qinghai-Tibetan Plateau. Our main objectives were to identify the potential effects of grazing intensity on alpine grasslands on the Qinghai-Tibetan Plateau. The findings will help guide sustainable grassland management for alpine rangeland ecosystems.

## 2 Materials and methods

### 2.1 Data collection

**Figure 1:**
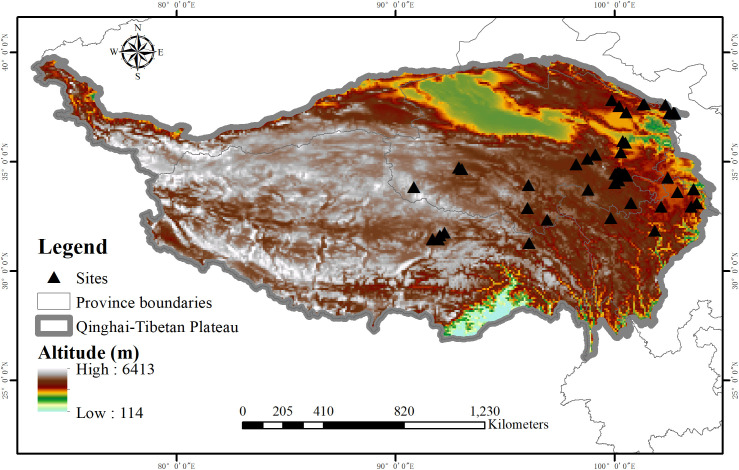
Distribution of alpine grassland sites across the Qinghai-Tibetan Plateau of the collected datasets in this study.

To construct a comprehensive database of grazing intensity effects on alpine grasslands, we collected peer-reviewed publications before July 2022 using the Web of Science (http://apps.webofknowledge.com/) and the China Knowledge Resource Integrated Database (http://www.cnki.net/). The searching term combinations were: “grazing or herbivory or fencing”, “alpine grassland or alpine steppe or alpine meadow”, and “Qinghai-Tibetan Plateau or Tibetan Plateau or Tibet”. Afterward, we screened the publications to identify appropriate studies based on the following criteria: (1) Only field experiments conducted in the alpine grassland of the Qinghai-Tibetan Plateau were included; (2) Grazing impacts were focused on alone without other confounding treatments (e.g. warming, precipitation change, or fertilization); (3) We also excluded simulated grazing experiments (e.g. mowing or trampling studies); (4) there was at least one pair of non-grazing (control group) and grazing (treatment group) treatments whose initial environmental and climate conditions, vegetation and soil types were the same; (5) Grazing intensity needs to be clearly described in each study; (6) Response variables were explicitly indicated by their means, standard deviation or standard error, and number of replicates. Totally, there were 52 published journal articles accorded with these criteria shown in the **Supplementary**, which included 38 alpine grassland sites across the Qinghai-Tibetan Plateau (**Figure 1**).

In the dataset, we collected 16 response variables of alpine grasslands which were divided into the following several categories: plant characteristics (plant coverage, aboveground biomass, belowground biomass, root: shoot, species richness, Shannon-Wiener index), soil physical properties (soil water content and soil bulk density), soil nutrients (soil organic carbon, total nitrogen, total phosphorus, available nitrogen, available phosphorus, ratio of carbon to nitrogen), and soil microbiomes (microbial biomass carbon and microbial biomass nitrogen). These response variables were all extracted directly from the body of the text, tables, or acquired from digitized graphs by using GetData Graph Digitizer software (ver. 2.25, www.getadata-graph-digitizer.com/). Additionally, the geographic coordinates (latitude and longitude) of each study were also recorded. According to Sun et al. (2021b), the grazing intensity was divided into light, moderate, and heavy grazing intensities considering utilization of grass, number of livestock, or distance from the source of water, as shown in **Table S1** in the **Supplementary**.

### 2.2 Data analyses

The meta-analysis was conducted by using the MetaWin 2.1 software (Sinauer Associates Inc., Sunderland, MA, USA) to determine whether different grazing intensities had significant effects on above- and belowground functions of alpine grasslands (Hedges et al., 1999). For all response variables, we calculated an effect size for the contrasts between no grazing with three grazing intensities (light, moderate and heavy). The effect size was estimated as the response ratio (*RR*) which was calculated as follows:


$$RR=\ln({\bar{X}}_{t}/{\bar{X}}_{c})$$

Where $\bar{\text{x}}$ _t_ and $\bar{\text{x}}$ _c_ are the arithmetic mean concentrations of the target variable in alpine grasslands with different grazing intensities (treatment group) and no grazing grasslands (control group), respectively.

The variance (*v*) of *RR* was estimated by the following equation:


$$v=\frac{S_{t}^{2}}{n_{t}{\bar{X}}_{t}^{2}}+\frac{S_{c}^{2}}{n_{c}{\bar{X}}_{c}^{2}}$$

where *n_t_* and *S_t_* are the sample sizes and the standard deviations of the target variable in the treatment group, respectively; *n_c_* and *S_c_* are the sample sizes and the standard deviations of the target variable the control group, respectively.

Then, the reciprocal of the variance (1/*v*) was used as the weight factor (*w*) for each *RR* value, which was further used to calculate the weighted response ratio (*RR*_++_) to improve the statistical accuracy. The mean response ratio (*RR*_++_) was calculated from individual *RR* values of each pairwise comparison between the no grazing and grazing group as follows:


$$RR_{++}=\frac{\sum_{i=1}^{m}\sum_{j=1}^{k}w_{ij}RR_{ij}}{\sum_{i=1}^{m}\sum_{j=1}^{k}w_{ij}}$$

where *m* and *k* are the number of treatment groups and the number of comparisons in the corresponding control group, respectively. *w*_ij_ and *RR*_ij_ are the weight factor and response ratio for each categorical group, respectively.

The 95% confidence interval (*CI*) values of *RR*_++_ were used to test the significance of grazing effect and were calculated as follows:


$$95\%CI=RR_{++}\pm 1.96S(RR_{++})$$

where S(RR++) is the standard error of RR++ which was estimated by the following equation:


$$S(RR_{++})=\sqrt{\frac{1}{\sum_{i=1}^{m}\sum_{j=1}^{k}w_{ij}}}$$

We applied the random-effects model to calculate the mean effect size for each study and derived the bootstrap 95% confidence interval (95% *CI*) for each categorical group *via* the bootstrapping method based on 5,000 iterations (Guo and Gifford, 2002; Janssens et al., 2010). It suggests a statistically significant response of the selected variables only if the 95% *CI* of the *RR*_++_ did not overlap with zero.

## 3 Results

### 3.1 Responses of plant characteristics to different grazing intensities

**Figure 2:**
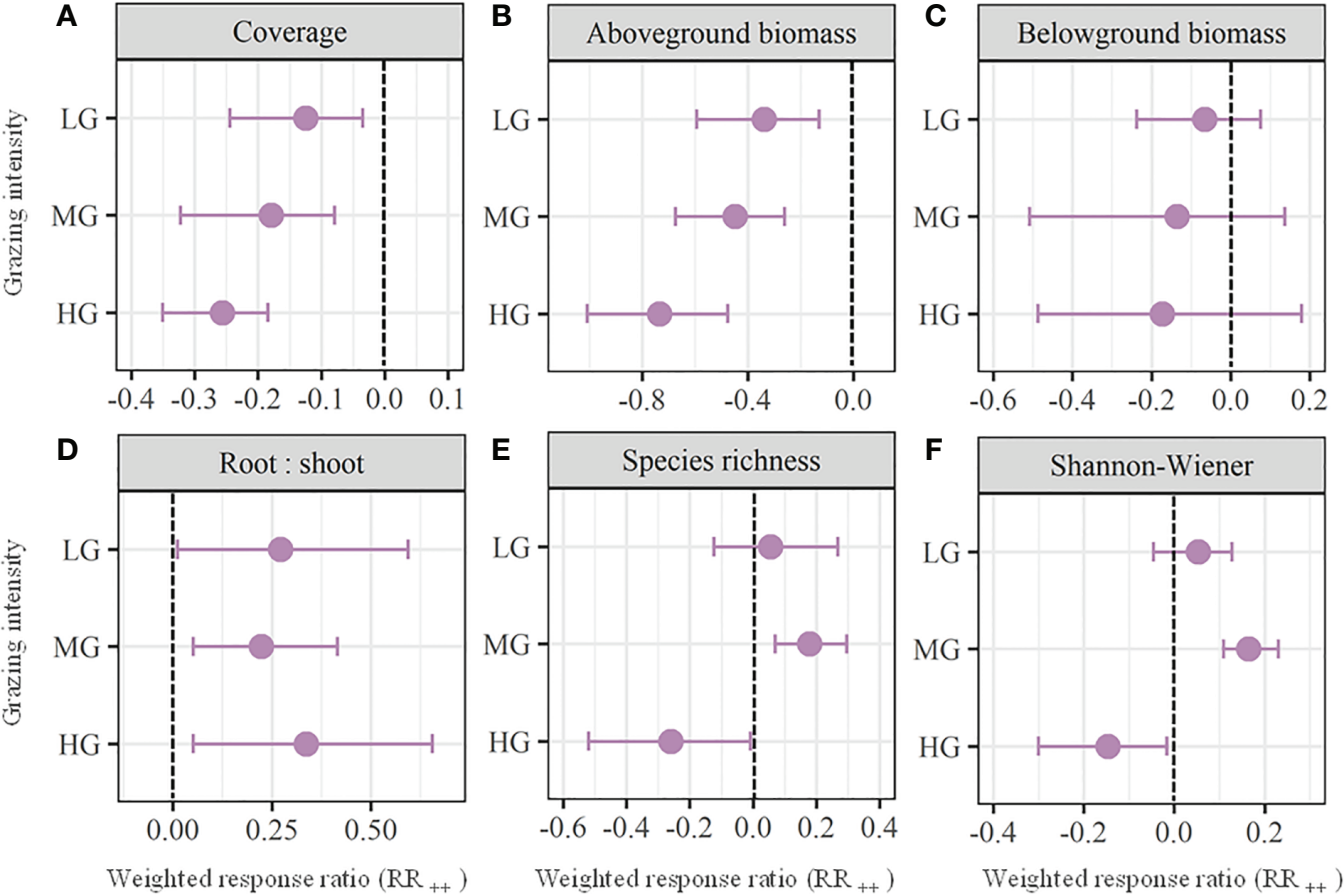
Weighted response ratios (RR_++_) and their 95% confidence intervals (CI) for plant characteristics including plant coverage **(A)**, aboveground biomass **(B)**, belowground biomass **(C)**, root: shoot **(D)**, species richness **(E)**, and Shannon-Wiener index **(F)** in the lightly (LD), moderately (MD), and heavily (HG) grazing alpine grasslands. The overall effect sizes are unitless grand means from weighted meta-analyses. The dots with error bars indicate the mean effect size with the 95% CI. The observed effect sizes were considered statistically significant if the 95% CI did not include zero.

Grazing significantly decreased the plant coverage and aboveground biomass compared with those of no grazing (*P<* 0.05; **Figures 2A, B**). Furthermore, the magnitude of reductions of both plant coverage and aboveground biomass gradually increased with increasing grazing intensity. Specifically, the mean weighted response ratios of plant coverage were -0.12, -0.18, and -0.26 for LG, MG, and HG, respectively; the mean weighted response ratios of aboveground biomass were -0.34, -0.45, and -0.73 for LG, MG, and HG, respectively. By contrast, grazing showed no significant effect on belowground biomass with all grazing intensities (*P* > 0.05; **Figure 2C**). Moreover, grazing significantly increased the root: shoot with the mean weighted response ratios of 0.27, 0.22, and 0.34 for LG, MG, and HG, respectively (**Figure 2D**). Additionally, both species richness and Shannon-Wiener index exhibited no significant changes with LG, while significantly increased with MG (RR_++_ = 0.18 and 0.16, respectively) and significantly decreased with HG (RR_++_ = -0.26 and -0.15, respectively; **Figures 2E, F**).

### 3.2 Responses of soil properties to different grazing intensities

**Figure 3:**
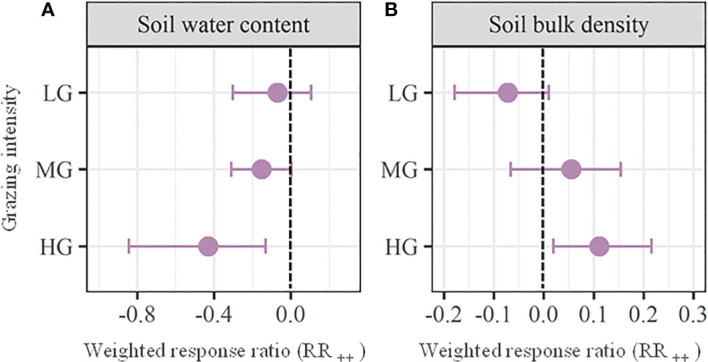
Weighted response ratios (RR_++_) and their 95% confidence intervals (CI) for soil physical properties including soil water content **(A)** and soil bulk density **(B)** in the lightly (LD), moderately (MD), and heavily (HG) grazing alpine grasslands. The overall effect sizes are unitless grand means from weighted meta-analyses. The dots with error bars indicate the mean effect size with the 95% CI. The observed effect sizes were considered statistically significant if the 95% CI did not include zero.

**Figure 4:**
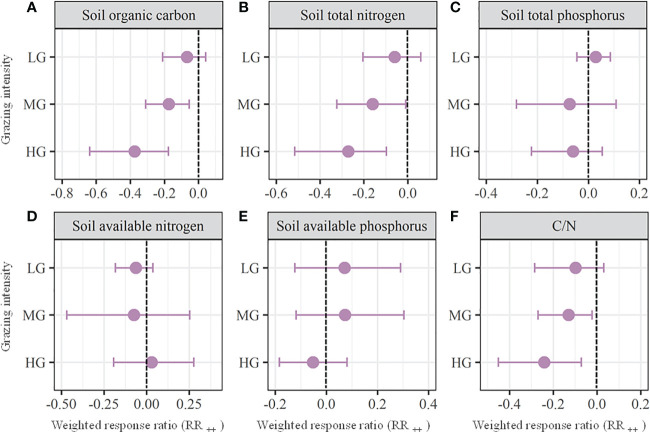
Weighted response ratios (RR_++_) and their 95% confidence intervals (CI) for soil nutrients including soil organic carbon **(A)**, total nitrogen **(B)**, total phosphorus **(C)**, available nitrogen **(D)**, available phosphorus **(E)**, and ratio of carbon to nitrogen **(F)** in the lightly (LD), moderately (MD), and heavily (HG) grazing alpine grasslands. The overall effect sizes are unitless grand means from weighted meta-analyses. The dots with error bars indicate the mean effect size with the 95% CI. The observed effect sizes were considered statistically significant if the 95% CI did not include zero.

**Figure 5:**
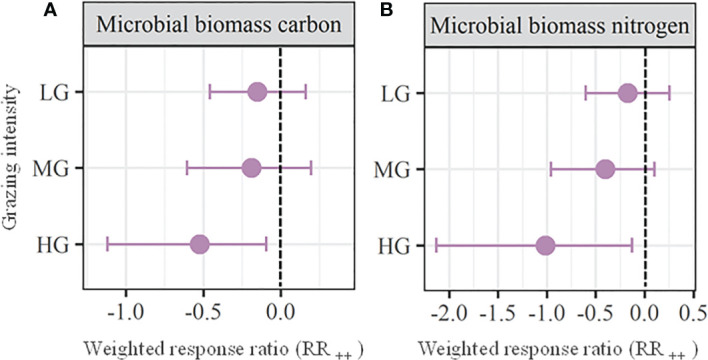
Weighted response ratios (RR_++_) and their 95% confidence intervals (CI) for soil microbiomes including microbial biomass carbon **(A)** and microbial biomass nitrogen **(B)** in the lightly (LD), moderately (MD), and heavily (HG) grazing alpine grasslands. The overall effect sizes are unitless grand means from weighted meta-analyses. The dots with error bars indicate the mean effect size with the 95% CI. The observed effect sizes were considered statistically significant ifthe 95% CI did not include zero.

We found that the soil water content (**Figure 3A**), soil bulk density (**Figure 3B**), soil total phosphorus (**Figure 4C**), available nitrogen (**Figure 4D**), available phosphorus (**Figure 4E**), microbial biomass carbon (**Figure 5A**) and microbial biomass nitrogen (**Figure 5B**) unchanged with MG (*P* > 0.05), which significantly decreased the soil organic carbon (RR_++_ = -0.17; **Figure 4A**), total nitrogen (RR_++_ = -0.16; **Figure 4B**), and ratio of carbon to nitrogen (RR_++_ = -0.13; **Figure 4F**). Across all the observations compiled in this study, our meta analysis showed that LG had no significant effects on all the above soil properties (*P* > 0.05; **Figures 3**-**5**). Similarly, HG did not significantly affect soil total phosphorus, available nitrogen, and available phosphorus (*P* > 0.05), while HG remarkably decreased soil water content (RR_++_ = -0.42), soil organic carbon (RR_++_ = -0.37), total nitrogen (RR_++_ = -0.27), ratio of carbon to nitrogen (RR_++_ = -0.24), microbial biomass carbon (RR_++_ = -0.52) and microbial biomass nitrogen (RR_++_ = -1.01) and significantly increase the soil bulk density (RR_++_ = 0.11) (**Figures 3**-**5**).

## 4 Discussion

### 4.1 Effects of grazing intensity on plant characteristics

Plant productivity serves as important metrics of ecosystem functions for grazing grasslands. In the current study, we found significant decreases in the plant aboveground biomass whose magnitudes gradually increased with increasing grazing intensity (**Figure 2**). It is due, for the most part, to the direct consumption and damage of plant tissue from large herbivores, and the extent of disturbance increases with grazing pressure (Lin et al., 2011; Deng et al., 2014). Previous studies revealed that the grazing-induced decrease in aboveground biomass could limit plant to make photosynthate which further inhibits root growth (Bagchi and Ritchie, 2010; Bai et al., 2015). However, the belowground biomass exhibited no significant change with all grazing intensities in this study (**Figure 2**). It might be that grazing induced plant compensatory effects which offset the negative effects on root growth (Bai et al., 2004; Niu et al., 2009). Consequently, the ratios of root to shoot were remarkably increased with all grazing intensities (**Figure 2**), which indicates that grazing leads to the biomass transfer from aboveground to belowground in alpine grasslands, in consistent with previous studies (De Deyn et al., 2008; Deng et al., 2014; Sun et al., 2021b).

Plant diversity is vital for maintaining grassland ecosystem function and stability (Chen et al., 2018; Pennekamp et al., 2018). Numerus studies have proven that the abundant plant aboveground biomass can exacerbate competition effects among young plant species and further result in loss of plant species in no grazing grasslands (Klein et al., 2004; Ruprecht et al., 2010; Zhang Z. C. et al., 2021). By contrast, the removal of plant aboveground biomass by herbivores contributes to apparent decreases in plant coverage (**Figure 2**) and thereby increases the reception of solar radiation by green plants and soil surface in the grazed pastures, which can increase soil temperature and promote germination rates and seedling survival (Wu et al., 2009; Tian et al., 2020). Therefore, we found moderate grazing intensity contributed to higher level of plant diversity in this study (**Figure 2**), which supports the intermediate disturbance hypothesis (McNaughton et al., 1997). However, once the disturbance exceeds a certain extent, heavy grazing intensity would show significantly negative effects on plant diversity (**Figure 2**), which was also revealed in numerus previous studies (Harris, 2010; Eldridge and Delgado-Baquerizo, 2017; Li et al., 2021).

### 4.2 Effects of grazing intensity on soil properties

The grazing-induced lower plant cover can not only increase the light availability at ground level, but also enhances soil evaporation due to exposing more soil into the air (Chen et al., 2011; Tian et al., 2016). As a result, we found that heavy grazing significantly decreased soil water content (**Figure 3**). Since soil water plays vital roles in the retention and transfer of available nutrients and multiple plant physiological activities (Liu et al., 2020; Zhou et al., 2020; Zhang et al., 2022), the intensified water limitation by heavy grazing will further suppress plant growth (Chen et al., 2011; Bagchi et al., 2017). Moreover, the soil bulk density was significantly increased by heavy grazing due to the trampling of large herbivores (**Figure 3**). The enhanced soil compaction can worsen the soil water and aeration conditions, which would further supress soil microbial activities (Holst et al., 2008; Chen et al., 2011). This explains the significant decreases in soil microbial biomass carbon and microbial biomass nitrogen with heavy grazing in this study (**Figure 5**).

For soil fertility, we found that both moderate and heavy grazing significantly decreased the soil carbon and nitrogen (**Figure 4**). It is mainly because of the suppressed organic matter accumulation and decomposition processes due to an outflow of nutrient from grassland to livestock as well as the suppressed soil microbial activities (Zhou et al., 2017; Liu et al., 2021; Wan et al., 2022). Moreover, the proportion of forbs with lower decomposability is generally improved due to the selective consumption of livestock, which can constrain the soil nutrient accumulation (Semmartin et al., 2010; Li et al., 2014; Zhang R. Y. et al., 2021). Previous studies found that the deposition of dung and urine from herbivores can improve soil nutrient availability (Kohler et al., 2005; Tian et al., 2021). However, the soil available nutrients unchanged with all grazing intensities in this study (**Figure 4**). The reason might be that the negative effects of decreased organic matter accumulation and decomposition offset the positive effects of dung and urine deposition. Also, the soil phosphorus unchanged with all grazing intensities in alpine grasslands (**Figure 4**). The logic might be that the soil phosphorus is mainly derived from rock weathering so as to be more responsive to parent materials and climate instead of grazing disturbance (Liu et al., 2021; Zhang Z. C. et al., 2021).

## 5 Conclusion

Our study reveals the impacts of different grazing intensities on plant characteristics and soil properties of alpine grasslands across the Qinghai-Tibetan Plateau. Specifically, we found that light grazing exhibited little effects on most plant characteristics and soil properties due to its light disturbance. Moderate grazing significantly improved plant diversity, while decreased soil nutrients due to an outflow of nutrient from grassland to livestock. Heavy grazing intensity not only weakens plant productivity and diversity, but also causes decreases in soil quality and fertility. These findings imply that overgrazing is closely related to alpine grassland degradation, while moderate grazing may be a sustainable practice to provide animal production and simultaneously maintain ecological functions for alpine grasslands. However, fertilization should be needed to keep soil fertility and grassland productivity for the moderate grazing ecosystems on the Qinghai-Tibetan Plateau. However, the effects of grazing intensity may vary with grassland types, grazing duration, and grazing management. Further studies to combine these factors are necessary for scientific assessments of effects of grazing on the alpine grasslands.

## Data availability statement

The original contributions presented in the study are included in the article/**Supplementary Material**. Further inquiries can be directed to the corresponding authors.

## Author contributions

ZZ and YaZ conceived the ideas and designed the methodology. ZZ, YiZ, YL, and YW collected and analyzed the data. ZZ, YiZ, and YL drew the graphs. ZZ, YiZ, YW, and YaZ wrote the manuscript. ZZ, YiZ, HL, YL, JF, YW, JS, and YaZ reviewed and revised the manuscript. All of the authors contributed critically to the drafts.

## References

[B1] Ameztegui A. Coll L. (2015). Herbivory and seedling establishment in pyrenean forests: Influence of micro- and meso-habitat factors on browsing pressure. For. Ecol. Manage. 342, 103–111. doi: 10.1016/j.foreco.2015.01.021

[B2] Bagchi S. Ritchie M. E. (2010). Introduced grazers can restrict potential soil carbon sequestration through impacts on plant community composition. Ecol. Lett. 13, 959–968. doi: 10.1111/j.1461-0248.2010.01486.x 20482575

[B3] Bagchi S. Roy S. Maitra A. Sran R. S. (2017). Herbivores suppress soil microbes to influence carbon sequestration in the grazing ecosystem of the trans-himalaya. Agricult. Ecosyst. Environ. 239, 199–206. doi: 10.1016/j.agee.2017.01.033

[B4] Bai W. M. Fang Y. Zhou M. Xie T. Li L. H. Zhang W. H. (2015). Heavily intensified grazing reduces root production in an inner Mongolia temperate steppe. Agricult. Ecosyst. Environ. 200, 143–150. doi: 10.1016/j.agee.2014.11.015

[B5] Bai Y. F. Han X. G. Wu J. G. Chen Z. Z. Li L. H. (2004). Ecosystem stability and compensatory effects in the inner Mongolia grassland. Nature 431, 181–184. doi: 10.1038/nature02850 15356630

[B6] Borer E. T. Seabloom E. W. Gruner D. S. Harpole W. S. Hillebrand H. Lind E. M.. (2014). Herbivores and nutrients control grassland plant diversity *via* light limitation. Nature 508, 517–520. doi: 10.1038/nature13144 24670649

[B7] Chen S. Wang W. Xu W. Wang Y. Wan H. Chen D.. (2018). Plant diversity enhances productivity and soil carbon storage. PNAS 115, 4027–4032. doi: 10.1073/pnas.1700298114 29666315 PMC5910804

[B8] Chen W. W. Wolf B. Zheng X. H. Yao Z. S. Butterbach-Bahl K. Bruggemann N.. (2011). Annual methane uptake by temperate semiarid steppes as regulated by stocking rates, aboveground plant biomass and topsoil air permeability. Global Change Biol. 17, 2803–2816. doi: 10.1111/j.1365-2486.2011.02444.x

[B9] Cui X. F. Graf H. (2009). Recent land cover changes on the Tibetan plateau: a review. Climatic Change 94, 47–61. doi: 10.1007/s10584-009-9556-8

[B10] De Deyn G. B. Cornelissen J. H. C. Bardgett R. D. (2008). Plant functional traits and soil carbon sequestration in contrasting biomes. Ecol. Lett. 11, 516–531. doi: 10.1111/j.1461-0248.2008.01164.x 18279352

[B11] Deng L. Shangguan Z. P. Wu G. L. Chang X. F. (2017). Effects of grazing exclusion on carbon sequestration in china's grassland. Earth Sci. Rev. 173, 84–95. doi: 10.1016/j.earscirev.2017.08.008

[B12] Deng L. Zhang Z. N. Shangguan Z. P. (2014). Long-term fencing effects on plant diversity and soil properties in China. Soil Tillage Res. 137, 7–15. doi: 10.1016/j.still.2013.11.002

[B13] Eldridge D. J. Delgado-Baquerizo M. (2017). Continental-scale impacts of livestock grazing on ecosystem supporting and regulating services. Land Degradation Dev. 28, 1473–1481. doi: 10.1002/ldr.2668

[B14] Farji-Brener A. G. Werenkraut V. (2017). The effects of ant nests on soil fertility and plant performance: A meta-analysis. J. Anim. Ecol. 86, 866–877. doi: 10.1111/1365-2656.12672 28369906

[B15] Fetzel T. Havlik P. Herrero M. Kaplan J. Kastner T. Kroisleitner C.. (2017). Quantification of uncertainties in global grazing systems assessment. Global Biogeochem. Cycles 31, 1089–1102. doi: 10.1002/2016GB005601

[B16] Guo L. B. Gifford R. M. (2002). Soil carbon stocks and land use change: a meta analysis. Global Change Biol. 8, 345–360. doi: 10.1046/j.1354-1013.2002.00486.x

[B17] Harris R. B. (2010). Rangeland degradation on the qinghai-Tibetan plateau: A review of the evidence of its magnitude and causes. J. Arid Environ. 74, 1–12. doi: 10.1016/j.jaridenv.2009.06.014

[B18] Hedges L. V. Gurevitch J. Curtis P. S. (1999). The meta-analysis of response ratios in experimental ecology. Ecology 80, 1150–1156. doi: 10.1890/0012-9658(1999)080[1150:TMAORR]2.0.CO;2

[B19] Holst J. Liu C. Yao Z. Brüggemann N. Zheng X. Giese M.. (2008). Fluxes of nitrous oxide, methane and carbon dioxide during freezing–thawing cycles in an inner Mongolian steppe. Plant Soil 308, 105–117. doi: 10.1007/s11104-008-9610-8

[B20] Janssens I. A. Dieleman W. Luyssaert S. Subke J. A. Reichstein M. Ceulemans R.. (2010). Reduction of forest soil respiration in response to nitrogen deposition. Nat. Geosci. 3, 315–322. doi: 10.1038/ngeo844

[B21] Kemp D. R. Han G. Hou X. Michalk D. L. Hou F. Wu J.. (2013). Innovative grassland management systems for environmental and livelihood benefits. Proc. Natl. Acad. Sci. 110, 8369–8374. doi: 10.1073/pnas.1208063110 23671092 PMC3666733

[B22] Klein J. A. Harte J. Zhao X. Q. (2004). Experimental warming causes large and rapid species loss, dampened bysimulated grazing, on the Tibetan plateau. Ecol. Lett. 7, 1170–1179. doi: 10.1111/j.1461-0248.2004.00677.x

[B23] Kohler F. Hamelin J. Gillet F. Gobat J. M. Buttler A. (2005). Soil microbial community changes in wooded mountain pastures due to simulated effects of cattle grazing. Plant Soil 278, 327–340. doi: 10.1007/s11104-005-8809-1

[B24] Li Y. Y. Dong S. K. Wen L. Wang X. X. Wu Y. (2014). Soil carbon and nitrogen pools and their relationship to plant and soil dynamics of degraded and artificially restored grasslands of the qinghai–Tibetan plateau. Geoderma 213, 178–184. doi: 10.1016/j.geoderma.2013.08.022

[B25] Li W. L. Liu C. L. Wang W. Zhou H. K. Xue Y. T. Xu J.. (2021). Effects of different grazing disturbances on the plant diversity and ecological functions of alpine grassland ecosystem on the qinghai-Tibetan plateau. Front. Plant Sci. 12, 765070. doi: 10.3389/fpls.2021.765070 34966399 PMC8710682

[B26] Lin X. W. Zhang Z. H. Wang S. P. Hu Y. G. Xu G. P. Luo C. Y.. (2011). Response of ecosystem respiration to warming and grazing during the growing seasons in the alpine meadow on the Tibetan plateau. Agric. For. Meteorol. 151, 792–802. doi: 10.1016/j.agrformet.2011.01.009

[B27] Liu C. L. Li W. L. Xu J. Wei W. Xue P. F. Yan H. P. (2021). Response of soil nutrients and stoichiometry to grazing management in alpine grassland on the qinghai-Tibet plateau. Soil Tillage Res. 206, 104822. doi: 10.1016/j.still.2020.104822

[B28] Liu M. Zhang Z. C. Sun J. Wang Y. Wang J. N. Tsunekawa A.. (2020). One-year grazing exclusion remarkably restores degraded alpine meadow at zoige, eastern Tibetan plateau. Global Ecol. Conserv. 22, e00951. doi: 10.1016/j.gecco.2020.e00951

[B29] McNaughton S. J. Banyikwa F. F. McNaughton M. M. (1997). Promotion of the cycling of dietenhancing nutrients by African grazers. Science 278, 1798–1800. doi: 10.1126/science.278.5344.1798 9388182

[B30] Niu K. C. Choler P. Zhao B. B. Du G. Z. (2009). The allometry of reproductive biomass in response to land use in Tibetan alpine grasslands. Funct. Ecol. 23, 274–283. doi: 10.1111/j.1365-2435.2008.01502.x

[B31] Pennekamp F. Pontarp M. Tabi A. Altermatt F. Alther R. Choffat Y.. (2018). Biodiversity increases and decreases ecosystem stability. Nature 563, 109–112. doi: 10.1038/s41586-018-0627-8 30333623

[B32] Piao S. L. Tan K. Nan H. J. Ciais P. Fang J. Y. Wang T.. (2012). Impacts of climate and CO2 changes on the vegetation growth and carbon balance of qinghai-Tibetan grasslands over the past five decades. Global Planetary Change 98, 73–80. doi: 10.1016/j.gloplacha.2012.08.009

[B33] Ruprecht E. Enyedi M. Z. Eckstein R. L. Donath T. W. (2010). Restorative removal of plant litter and vegetation 40 years after abandonment enhances re-emergence of steppe grassland vegetation. Biol. Conserv. 143, 449–456. doi: 10.1016/j.biocon.2009.11.012

[B34] Sanjari G. Ghadiri H. Ciesiolka C. A. A. Yu B. F. (2008). Comparing the effects of continuous and time-controlled grazing systems on soil characteristics in southeast Queensland. Aust. J. Soil Res. 46, 348–358. doi: 10.1071/SR07220

[B35] Semmartin M. A. Bella C. Salamone I. S. (2010). Grazing-induced changes in plant species composition affect plant and soil properties of grassland mesocosms. Plant Soil 328, 471–481. doi: 10.1007/s11104-009-0126-7

[B36] Steffens M. Kolbl A. Kogel-Knabner I. (2010). Alteration of soil organic matter pools and aggregation in semi-arid steppe topsoils as driven by organic matter input. Eur. J. Soil Sci. 60, 198–212. doi: 10.1111/j.1365-2389.2008.01104.x

[B37] Sun J. Fu B. J. Zhao W. W. Liu S. L. Zhou H. K. Shao X. Q.. (2021a). Optimizing grazing exclusion practices to achieve goal 15 of the sustainable development goals in the Tibetan plateau. Sci. Bull. 66, 1493–1496. doi: 10.1016/j.scib.2021.03.014 36654275

[B38] Sun J. Liu M. Fu B. J. Kemp D. Zhao W. W. Liu G. H.. (2020). Reconsidering the efficiency of grazing exclusion using fences on the Tibetan plateau. Sci. Bull. 65, 1405–1414. doi: 10.1016/j.scib.2020.04.035 36659220

[B39] Sun J. Ma B. B. Lu X. Y. (2018). Grazing enhances soil nutrient effects: Trade-offs between aboveground and belowground biomass in alpine grasslands of the Tibetan plateau. Land Degradation Dev. 29, 337–348. doi: 10.1002/ldr.2822

[B40] Sun J. Qin X. J. (2016). Precipitation and temperature regulate the seasonal changes of NDVI across the Tibetan plateau. Environ. Earth Sci. 75, 291. doi: 10.1007/s12665-015-5177-x

[B41] Sun J. Wang H. M. (2016). Soil nitrogen and carbon determine the trade-off of the above- and below-ground biomass across alpine grasslands, Tibetan plateau. Ecol. Indic. 60, 1070–1076. doi: 10.1016/j.ecolind.2015.08.038

[B42] Sun J. Zhan T. Y. Liu M. Zhang Z. C. Wang Y. Liu S. L.. (2021b). Verification of the biomass transfer hypothesis under moderate grazing across the Tibetan plateau: a meta-analysis. Plant Soil 458, 139–150. doi: 10.1007/s11104-019-04380-8

[B43] Tian L. H. Bai Y. F. Wang W. W. Qu G. P. Zhao J. X. (2021). Warm- and cold- season grazing affect plant diversity and soil carbon and nitrogen sequestration differently in Tibetan alpine swamp meadows. Plant Soil 458, 151–164. doi: 10.1007/s11104-020-04573-6

[B44] Tian L. H. Bai Y. F. Wang W. W. Qu G. P. Zhao J. H. Li R. C.. (2020). Warm- and cold- season grazing affect plant diversity and soil carbon and nitrogen sequestration differently in Tibetan alpine swamp meadows. Plant Soil 458, 151–164. doi: 10.1007/s11104-020-04573-6

[B45] Tian D. S. Niu S. L. Pan Q. M. Ren T. T. Chen S. P. Bai Y. F.. (2016). Nonlinear responses of ecosystem carbon fluxes and water-use efficiency to nitrogen addition in inner Mongolia grassland. Funct. Ecol. 30, 490–499. doi: 10.1111/1365-2435.12513

[B46] Wan B. B. Liu T. Gong X. Zhang Y. Li C. J. Chen X. Y.. (2022). Energy flux across multitrophic levels drives ecosystem multifunctionality: Evidence from nematode food webs. Soil Biol. Biochem. 169, 108656. doi: 10.1016/j.soilbio.2022.108656

[B47] Wu G. L. Du G. Z. Liu Z. H. Thirgood S. (2009). Effect of fencing and grazing on a kobresia-dominated meadow in the qinghai-Tibetan plateau. Plant Soil 319, 115–126. doi: 10.1007/s11104-008-9854-3

[B48] Yang W. S. Liu Y. Zhao J. X. Chang X. F. Wiesmeier M. Sun J.. (2021). SOC changes were more sensitive in alpine grasslands than in temperate grasslands during grassland transformation in China: A meta-analysis. J. Cleaner Production 308, 127430. doi: 10.1016/j.jclepro.2021.127430

[B49] Yang Z. A. Wan X. Xu Y. Y. Jiang L. Zhu E. X. He X. W.. (2016). Soil properties and species composition under different grazing intensity in an alpine meadow on the eastern Tibetan plateau, China. Environ. Monit. Assess. 188, 678. doi: 10.1007/s10661-016-5663-y 27858261

[B50] Yan L. Li Y. Wang L. Zhang X. D. Wang J. Z. Wu H. D.. (2020). Grazing significantly increases root shoot ratio but decreases soil organic carbon in qinghai-Tibetan plateau grasslands: A hierarchical meta-analysis. Land Degradation Dev. 31, 2369–2378. doi: 10.1002/ldr.3606

[B51] Zhang Z. C. Liu Y. Sun J. Wu G. L. (2021). Suitable duration of grazing exclusion for restoration of a degraded alpine meadow on the eastern qinghai-Tibetan plateau. Catena 207, 105582. doi: 10.1016/j.catena.2021.105582

[B52] Zhang Z. C. Sun J. Liu M. Shang H. Wang J. N. Wang J. S.. (2022). Context-dependency in relationships between herbaceous plant leaf traits and abiotic factors. Front. Plant Sci. 13, 757077. doi: 10.3389/fpls.2022.757077 35401631 PMC8990845

[B53] Zhang R. Y. Wang Z. W. Niu S. L. Tian D. S. Wu Q. Gao X. F.. (2021). Diversity of plant and soil microbes mediates the response of ecosystem multifunctionality to grazing disturbance. Sci. Total Environ. 776, 145730. doi: 10.1016/j.scitotenv.2021.145730 33639460

[B54] Zhan T. Y. Zhang Z. C. Sun J. Liu M. Zhang X. B. Peng F.. (2020). Meta-analysis demonstrating that moderate grazing can improve the soil quality across china's grassland ecosystems. Appl. Soil Ecol. 147, 103438. doi: 10.1016/j.apsoil.2019.103438

[B55] Zhao J. X. Li X. Li R. C. Tian L. H. Zhang T. (2016). Effect of grazing exclusion on ecosystem respiration among three different alpine grasslands on the central Tibetan plateau. Ecol. Eng. 94, 599–607. doi: 10.1016/j.ecoleng.2016.06.112

[B56] Zhong Z. Li X. Sanders D. Liu Y. Wang L. Ortega Y. K.. (2021). Soil engineering by ants facilitates plant compensation for large herbivore removal of aboveground biomass. Ecology 102, e03312. doi: 10.1002/ecy.3312 33586130

[B57] Zhong Z. W. Li X. F. Smit C. Li T. Y. Wang L. Aschero V.. (2022). Large Herbivores facilitate a dominant grassland forb *via* multiple indirect effects. Ecology 103, e3635. doi: 10.1002/ecy.3635 35060616

[B58] Zhou T. C. Liu M. Sun J. Yurui L. Xue X. (2020). The patterns and mechanisms of precipitation use efficiency in alpine grasslands on the Tibetan plateau. Agric. Ecosyst. Environ. 2020, 106833. doi: 10.1016/j.agee.2020.106833

[B59] Zhou G. Y. Zhou X. H. He Y. H. Shao J. J. Hu Z. H. Liu R. Q.. (2017). Grazing intensity significantly affects belowground carbon and nitrogen cycling in grassland ecosystems: A meta-analysis. Global Change Biol. 23, 1167–1179. doi: 10.1111/gcb.13431 27416555

