# Supplementary material for: Comprehensive analysis of grazing intensity impacts alpine grasslands across the Qinghai-Tibetan Plateau: A meta-analysis
Source: Front Plant Sci. 2023 Jan 17;13:1083709. doi: 10.3389/fpls.2022.1083709 (PMC9887153; doi:10.3389/fpls.2022.1083709)
Supplement: Supplementary file 1 [file Table_1.docx]

Supplementary Material

# List of all the references used in the meta-analysis

Chen, D. D., Sun, D. S., Zhang, S. H., Tan, Y. R., Du, G. Z., and Shi, X. M. (2011). Effect of grazing intensity on soil microbial characteristics of an alpine meadow on the tibetan plateau. Journal of Lanzhou University(Natural Sciences) 47, 73-77.

Cui, S., Zhu, X., Wang, S., Zhang, Z., Xu, B., Luo, C. et al. (2014). Effects of seasonal grazing on soil respiration in alpine meadow on the Tibetan plateau. Soil Use & Management 30, 435-443.

Dai, L. C., Guo, X. W., Ke, X., Zhang, F. W., Li, Y. K., Peng, C. J. et al. (2019). Moderate grazing promotes the root biomass in Kobresia meadow on the northern Qinghai–Tibet Plateau. Ecology and Evolution 9, 9395-9406.

Dong, Q. M., Zhao, X. Q., Wu, G. L., Shi, J. J., and Sheng, L. (2012). Response of soil properties to yak grazing intensity in a Kobresia parva-meadow on the Qinghai-Tibetan Plateau, China. Journal of Soil Science & Plant Nutrition 12, 535-546.

Duan, M. J., Gao, Q. Z., Wan, Y. F., Li, Y., Guo, Y. Q., Danjiu, L. B. et al. (2010). Effect of grazing on community characteristics and species diversity of *Stipa purpurea* alpine grassland in Northern Tibet. Acta Ecologica Sinica 30, 3892-3900.

Duan, M. J., Gao, Q. Z., Wan, Y. F., Li, Y., Guo, Y. Q., Ganzhu, Z. B. et al. (2011). Biomass estimation of alpine grasslands under different grazing intensities using spectral vegetation indices. Canadian Journal of Remote Sensing 37, 413-421.

Fu, G., Shen, Z. X., Zhang, X. Z., Zhou, Y. T., and Zhang, Y. J. (2012). Response of microbial biomass to grazing in an alpine meadow along an elevation gradient on the Tibetan Plateau. European Journal of Soil Biology 52, 27-29.

Fu, g., Zhang, X. Z., Yu, C. Q., Shi, P. L., Zhou, Y. T., Li, Y. L. et al. (2014). Response of soil respiration to grazing in an alpine meadow at three elevations in Tibet. The Scientific World Journal 265142, 1-9.

Fu, J. J., Yi, X. C. M., Chen, H., Miao, Y. J., and Hu, T. M. (2013). Responses of dominant plant nutrients to grazing intensity in Kobresia pygmaea meadow of the Qinghai-Tibet plateau. Pratacultural Science 30, 560-565.

Gong, Y. M., Mohammat, A., Liu, X. J., Li, K. H., Christie, P., Fang, F. et al. (2014). Response of carbon dioxide emissions to sheep grazing and N application in an alpine grassland – Part 1: Effect of sheep grazing. Biogeosciences 11, 1743-1750.

He, Y. G., Sun, H. Z., Shi, X. M., Qi, W., and Du, G. Z. (2015). Soil properties of Tibetan Plateau alpine wetland affected by grazing and season. Acta Prataculturae Sinica 24, 12-20.

Li, H. Q., Wei, Y. X., He, H. D., Yang, Y. S., and Li, Y. N. (2018). Effects of grazing density on nitrous oxide effluxes in alpine Kobresia Humilis meadow on the Qinghai-Tibetan Plateau. Chinese Journal of Agrometeorology 39, 27-33.

Li, S. Q., Wang, X. Z., Guo, Z. G., Zhou, J., Xue, R., and Shen, Y. Y. (2013). Effects of short-term grazing on C and N content in soil and soil microbe in alpine meadow in the north-eastern edge of the Qinghai-Tibetan Plateau. Chinese Journal of Grassland 35, 55-60+66.

Li, W., Cao, W. X., Li, X. L., Xu, C. L., and Shi, S. L. (2016a). Effect of different grazing management on soil nutrient characteristcs in alpine meadow-steppe. Grassland and Turf 36, 8-13.

Li, W., Cao, W. X., Shi, S. L., Li, X. L., Chen, J. G., and Xu, C. L. (2016b). Changes in organic carbon and nitrogen storage in alpine meadows under different grazing management regimes. Prataculturae Sinica 25, 25-33.

Lin, B., Zhao, X. R., Zheng, Y., Qi, S., and Liu, X. Z. (2017). Effect of grazing intensity on protozoan community, microbial biomass, and enzyme activity in an alpine meadow on the Tibetan Plateau. Journal of Soil and Sediments 17, 2752-2762.

Lin, X. W., Zhang, Z. H., Wang, S. P., Hu, Y. G., Xu, G. P., Luo, C. Y. et al. (2011). Response of ecosystem respiration to warming and grazing during the growing seasons in the alpine meadow on the Tibetan plateau. Agricultural & Forest Meteorology 151, 792-802.

Liu, Y. W., Tenzintarchen, Geng, X. D., Wei, D., Dai, D. X., and Xu-Ri (2020). Grazing exclusion enhanced net ecosystem carbon uptake but decreased plant nutrient content in an alpine steppe. Catena 195, 104799.

Liu, Z., Chen D. D., Li, Q., Zhao, L., Xu S. X., and Zhao, X. Q. (2016). Effects of different land use patterns on soil inorganic carbon in alpine meadow ecosystem. Bulletin of Soil and Water Conservation 36, 73-79.

Luan, J. W., Cui, L. J., Xiang, C. H., Wu, J. H., Song, H. T., Ma, Q. F. et al. (2014). Different grazing removal exclosures effects on soil C stocks among alpine ecosystems in east Qinghai–Tibet Plateau. Ecological Engineering 64, 262-268.

Luo, C., Xu, G., Chao, Z., Wang, S., Lin, X., Hu, Y. et al. (2010). Effect of warming and grazing on litter mass loss and temperature sensitivity of litter and dung mass loss on the Tibetan plateau. Global Change Biology 16, 1606-1617.

Luo, C. Y., Xu, G. P., Wang, Y. F., Wang, S. P., Lin, X. W., Hu, Y. G. et al. (2009). Effects of grazing and experimental warming on DOC concentrations in the soil solution on the Qinghai-Tibet plateau. Soil Biology & Biochemistry 41, 2493-2500.

Mao, S. J., Wu, Q. H., Li, H. Q., Zhang, F. W., and Li, Y. N. (2015). Effects of grazing intensity on species diversity and biomass in alpine-cold forb meadow on the Tibetan Plateau. Journal of Glaciology and Geocryology 37, 1372-1380.

Miao, Y. J., Fu, J. J., Sun, Y. F., Zhu, X. T., Yi, X. C. M., Hu, T. M. et al. (2014). Effects of yaks grazing methods on the community characteristics of Kobresia pygmaea meadow in Tibet. Acta Agrestia Sinica 22, 935-941.

Mipam, T. D., Chen, S. Y., Liu, J. Q., Miehe, G., and Tian, L. M. (2021). Short-term yak-grazing alters plant-soil stoichiometric relations in an alpine meadow on the eastern Tibetan Plateau. Plant and Soil 458, 125-137.

Ren, L., Yuan, Z. R., Chen, J. G., Li, S., Zhang, D. G., and Lin, D. (2016). Characteristics of soil nutrients in alpine meadow under different utilization patterns in eastern qilian mountains. Journal of Gansu Agricultural University 51, 70-75.

Rui, Y., Wang, S., Xu, Z., Wang, Y., Chen, C., Zhou, X. et al. (2011). Warming and grazing affect soil labile carbon and nitrogen pools differently in an alpine meadow of the Qinghai–Tibet Plateau in China. Journal of Soils and Sediments 11, 903-914.

Sheng, H. Y., Zhang, C. P., Cao, G. M., and Zhu, G. F. (2009). Effect of grazing on soil environment of alpine meadow dominated by Potentilla froticosa shrub on Qilian Mountain. Ecology and Environmental Sciences, 18, 1088-1093.

Su, S. L., Li, Y., Wang, L. Y., Guo, D., Kang, H. J., Li, X. D. et al. (2014). Effect of fencing on plant biomass and functional group structure of different types of degraded grassland in Qinghai-Tibet Plateau. Acta Botanica Boreali-occidentalia Sinica 34, 1652-1657.

Su, Z. S., Sun, Y. F., Fu, J. J., Chu, X. T., Xu, Y. F., and Hu, T. M. (2015). Effects of grazing intensity on soil nutrient of Kobresiapygmaea meadow in Tibet Plateau. Pratacultural Science 32, 322-328.

Sun, G., Zhu-Barker, X., Chen, D. M., Liu, L., Zhang, N. N., Shi, C. G. et al. (2017). Responses of root exudation and nutrient cycling to grazing intensities and recovery practices in an alpine meadow: An implication for pasture management. Plant & Soil 416, 515-525.

Sun, J., Wang, X., Cheng, G., Wu, J., and Hong, J. (2014). Effects of grazing regimes on plant traits and soil nutrients in an alpine steppe. Plos One 9, e108821.

Suo, C. X., Fei, X., Liu, Y. Z., Xiang, S., and Sun, S. C. (2022). Functional group characteristics of plant community at different grazing intensities in alpine grassland of northwestern Sichuan. Chinese Journal of Applied and Environmental Biology Doi: 10.19675/j.cnki.1006-687x.2021.09044.

Tan, Y. R., Du, G. Z., Chen, D. D., and Sun, D. S. (2012). Impact of grazing on the activities of soil enzymes and soil nutrient factors in an alpine meadow on the Qinghai-Tibetan plateau. Journal of Lanzhou University(Natural Sciences) 48, 86-91.

Tserang, D. K., Wen, L. Y., Ai, Y., Zhao, H. W., and Chen, Y. J. (2016). Impact of different grazing intensity on soil physical properties and plant biomass in Qinghai-Tibet plateau alpine meadow ecosystem. Pratacultural Science 33, 1975-1980.

Wang, M. M., Wang, S. P., Wu, L. W., Xu, D. P., Lin, Q. Y., Hu, Y. G. et al. (2016). Evaluating the lingering effect of livestock grazing on functional potentials of microbial communities in Tibetan grassland soils. Plant & Soil 407, 385-399.

Wang, Q. L., Wang, C. T., Du, Y. G., and Cao, G. M. (2008). Grazing impact on soil microbial biomass carbon and relationships with soil environment in alpine Kobresia meadow. Acta Prataculturae Sinica 17, 39-46.

Wang, X. D., Yan, Y., and Cao, Y. Z. (2012). Impact of historic grazing on steppe soils on the northern Tibetan Plateau. Plant & Soil 354, 173-183.

Wang, X. T., Zhang, S. H., Chen, D. D., Dan, Y. R., Sun, D. S., and Du, G. Z. (2010). The effects of natural grazing intensity on plant community and soil nutrients in alpine meadow. Acta Agrestia Sinica 18, 510-516.

Wei, Y. L., Cao, W. X., and Liu, Y. Z. (2018). Effect of grazing intensity and fencing on soil microbial biomass in alpine shrubland. Grassland and Turf 38, 3-9.

Wu, G. L., Li, X. P., Cheng, J. M., Wei, X. H., and Sun, L. (2009). Grazing disturbances mediate species composition of alpine meadow based on seed size. Israel Journal of Ecology & Evolution 55, 369-379.

Wu, G. L., Liu, Z. H., Lei, Z., Chen, J. M., and Hu, T. M. (2010). Long-term fencing improved soil properties and soil organic carbon storage in an alpine swamp meadow of western China. Plant & Soil 332, 331-337.

Xie, Z., Rou, X. L., Wang, C., Gu, Z., An, M., Nan, H. et al. (2014). Identifying response groups of soil nitrifiers and denitrifiers to grazing and associated soil environmental drivers in Tibetan alpine meadows. Soil Biology & Biochemistry 77, 89-99.

Yang, H. S., Bater, N., Zhou, X. H., Miao, X. L., Su, X. C., and Chang, G. Z. (2009). Influence of different grazing intensity to soil fertility in the subei alpine steppes. Journal of Soil and Water Conservation 23, 150-153.

Yang, Z., Zhu, Q., Zhan, W., Xu, Y., Zhu, E., Gao, Y. et al. (2018). The linkage between vegetation and soil nutrients and their variation under different grazing intensities in an alpine meadow on the eastern Qinghai-Tibetan Plateau. Ecological Engineering 110, 128-136.

Yang, Z. A., Xiong, W., Xu, Y. Y., Jiang, L., and Chen, H. (2016). Soil properties and species composition under different grazing intensity in an alpine meadow on the eastern Tibetan Plateau, China. Environmental Monitoring and Assessment 188, 678.

Yi, X. C. M., Xu, Y. F., Fu, J. J., Sun, Y. F., Sang, B. J. B., Ni, B. et al. (2014). Effects of grazing intensity on vegetation community and soil physicochemical properties of alpine meadow in Tibet. Journal of Northwest A & F University 42, 27-33.

Zhai, W. T., Chen, D. D., Li, Q., Zhao, L., Liu, Z., Xu, S. X. et al. (2017). Effect of grazing intensity on carbon metabolic characteristics of soil microbial communities in an alpine steppe in the regions around Qinghai Lake. Chinese Journal of Applied & Environmental Biology 23, 685-692.

Zhang, Y., Gao, Q., Dong, S., Liu, S., Wang, X., Su, X. et al. (2015). Effects of grazing and climate warming on plant diversity, productivity and living state in the alpine rangelands and cultivated grasslands of the Qinghai-Tibetan Plateau. Rangeland Journal 37, 57-65.

Zhao, N., Zhang, H. X., Wang, Ｒ. M., Yang, M. Y., Zhang, Y., Zhao, X. N. et al. (2014). Effect of grazing intensity on temperature sensitivity of soil nitrogen mineralization in Zoigё alpine meadow. Acta Ecologica Sinica 34, 4234-4241.

# Supplementary Tables

**Table S1.** Classification of grazing intensity

| Grazing intensity | Utilization of forage grass (%) | Number of sheep (hm^-2^) | Number of yak (hm^-2^) | Distance from the source of water |
| --- | --- | --- | --- | --- |
|  |  |  |  |  |
| Non-grazed | 0 | 0 | 0 | - |
| Light grazing | 0-30 | 0-5 | 0-1.25 | Far |
| Moderate grazing | 30-60 | 5-10 | 1.25-2.5 | Medium |
| Heavy grazing | > 60 | > 10 | > 2.5 | Nearby |
